# Novel Local Antifungal Treatment for Fungal Periprosthetic Joint Infection With Continuous Local Antibiotic Perfusion: A Surgical Technique

**DOI:** 10.1016/j.artd.2023.101245

**Published:** 2023-11-07

**Authors:** Hyonmin Choe, Akihiro Maruo, Yuta Hieda, Koki Abe, Naomi Kobayashi, Hiroyuki Ike, Ken Kumagai, Masanobu Takeyama, Yusuke Kawabata, Yutaka Inaba

**Affiliations:** a Department of Orthopaedic Surgery, Yokohama City University, Yokohama, Kanagawa, Japan; b Department of Orthopaedic Surgery, Harima Himeji General Medical Center, Himeji, Japan; c Department of Orthopaedic Surgery, Yokohama City University Medical Center, Yokohama, Kanagawa, Japan

**Keywords:** Antibiotic perfusion, Fungal, Periprosthetic joint infection

## Abstract

Fungal periprosthetic joint infections are one of the most intractable orthopedic disorders. Continuous local antibiotic perfusion allows direct administration of the antifungal agent micafungin into the local infection area at biofilm-disruptive concentrations, while controlling the dead space in addition to conventional treatment. Although the appropriate use of continuous local antibiotic perfusion requires familiarity with the characteristics of local antibiotic perfusion, it is a versatile treatment modality that can improve the clinical outcomes of fungal periprosthetic joint infection in combination with conventional treatment methods.

## Introduction

Periprosthetic joint infections (PJI) are one of the most intractable complications of orthopedic diseases. Bacteria that form biofilms around implants or in bone and soft tissues are known to have very different characteristics compared with floating bacteria that can be identified in isolated cultures [1]. As biofilms protect internal bacteria from antimicrobial agents and immune responses, systemic antimicrobial administration based on the minimum inhibitory concentration is frequently ineffective in PJI [1], and the surgical removal of infected implants is common with extensive debridement [2]. Several antimicrobial agents have been reported to destroy biofilms upon exposure to high concentrations for certain periods [3,4]. Recently, the antibiotic concentration required to destroy biofilms was defined as the minimum biofilm eradication concentration (MBEC) [1,3]; however, the MBEC was reported to be much higher than the minimum inhibitory concentration [[3], [4], [5], [6], [7]].

As well as bacterial species, fungi also cause PJI, and fungal PJI is known to have poorer postoperative outcomes than bacterial PJI because of the large loss of normal tissue, widespread infection, multiple surgical histories, and coinfection with other bacteria [[8], [9], [10], [11], [12], [13]]. Antimicrobial agents loaded with orthopedic bone cement (polymethylmethacrylate [PMMA]) have been reported to be effective against fungal PJI [14]. The disadvantages of PMMA include the inactivation of antimicrobials owing to polymerization heat and the limited release period of antimicrobials [4,15]. While there are heat-stable agents that can be effective in the setting of fungal PJI, the need for the agent to be even more heat stable limits the anti-fungal agents available for use. There is currently only one option for local antimicrobial treatment in patients with PJIs treated with debridement, antibiotics, or implant retention (DAIR). In addition, despite 2-staged surgery, patients often experience recurrence of local infection after revision total hip arthroplasty (THA) for fungal PJI [9,10,14]. Effective local antifungal treatment with revision THA or DAIR may result in better treatment outcomes for patients with fungal PJI.

Continuous local antibiotic perfusion (CLAP) locally administers high concentrations of antimicrobials. It was first induced in fracture-related infections caused by orthopedic implants [16,17]. CLAP enables the perfusion of high concentrations of antimicrobials based on MBEC at low flow rates into the bone marrow (intra-medullary antibiotics perfusion [iMAP]) or into the soft tissues (intra-soft tissue antibiotics perfusion [iSAP]) and joints (intra-joint antibiotics perfusion [iJAP]) while aspirating the synovial fluid, hematomas, and outdated antimicrobials using a combination of negative pressure wound therapy (NPWT) and drainage tubes [16]. Thus, CLAP can be useful for fungal PJI by perfusing active antifungals directly into the local infection site while controlling dead spaces [18,19]. We experienced successful CLAP in 4 cases of fungal PJI, including 2 fungal PJI cases in which implant retention was possible. Herein, we describe a detailed surgical technique and local antifungal treatment using CLAP with micafungin (MCFG) for fungal PJI.

## Surgical technique

### Surgical plan

**Figure 1:**
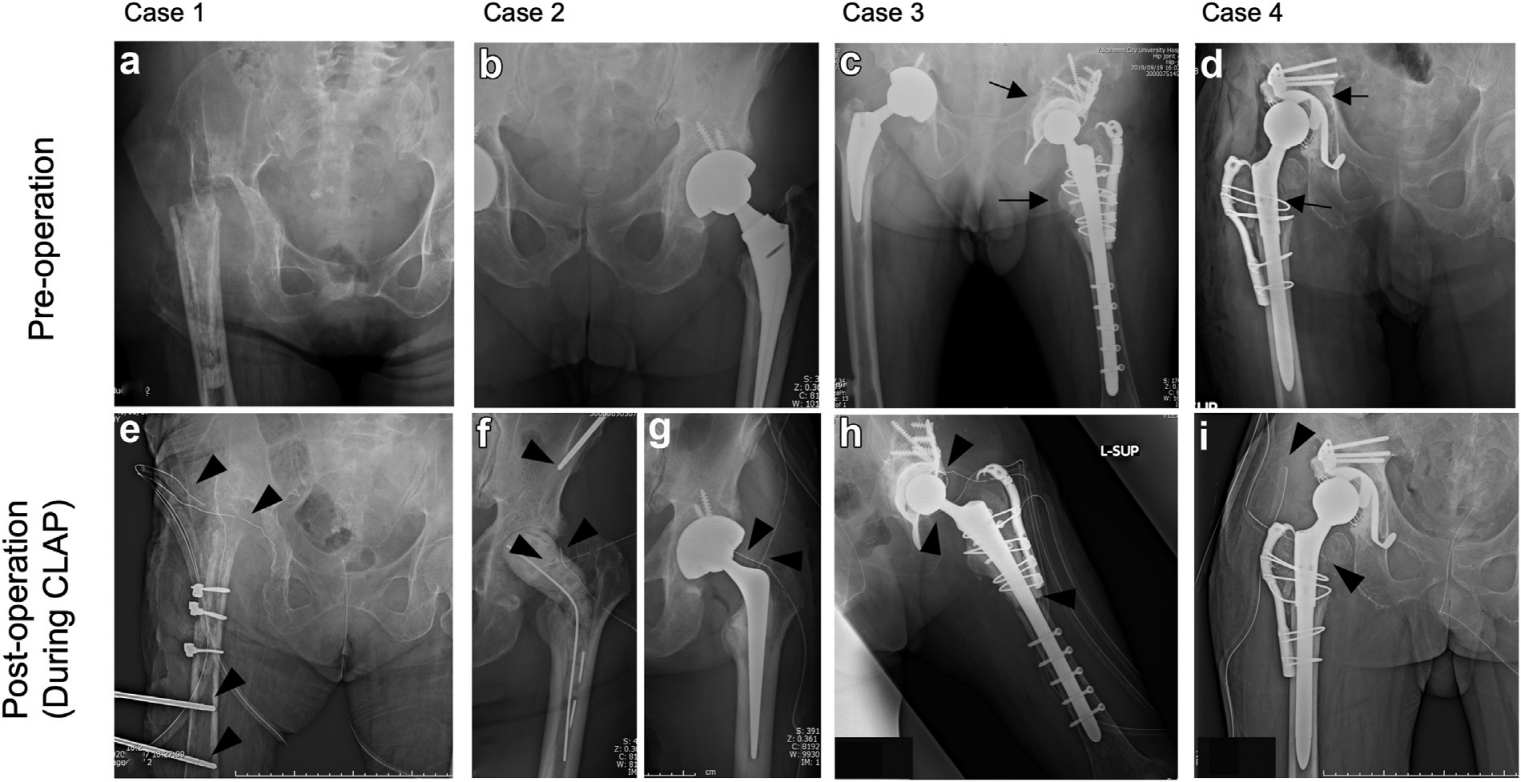
Preoperative and postoperative radiography in fungal periprosthetic joint infections (PJI). (a-d) Preoperative radiography for fungal PJI cases including patients with resection arthroplasty with massive bone defects (a, case 1), septic implant loosening (b, case 2), and patients after revision total hip arthroplasty (THA) with a support cage and cable plate with allogeneic bone grafts (arrows in c and d, cases 3 and 4). (e-i) Salem Sump Tubes were used in combination with iMap pins in femur and acetabulum (arrow head in e and f) to perfuse micafungin to infected site in debridement (e, case 1), 2-stage revision THA (f, case 2), and implant retention (h and i, cases 3 and 4) cases.

**Figure 2:**
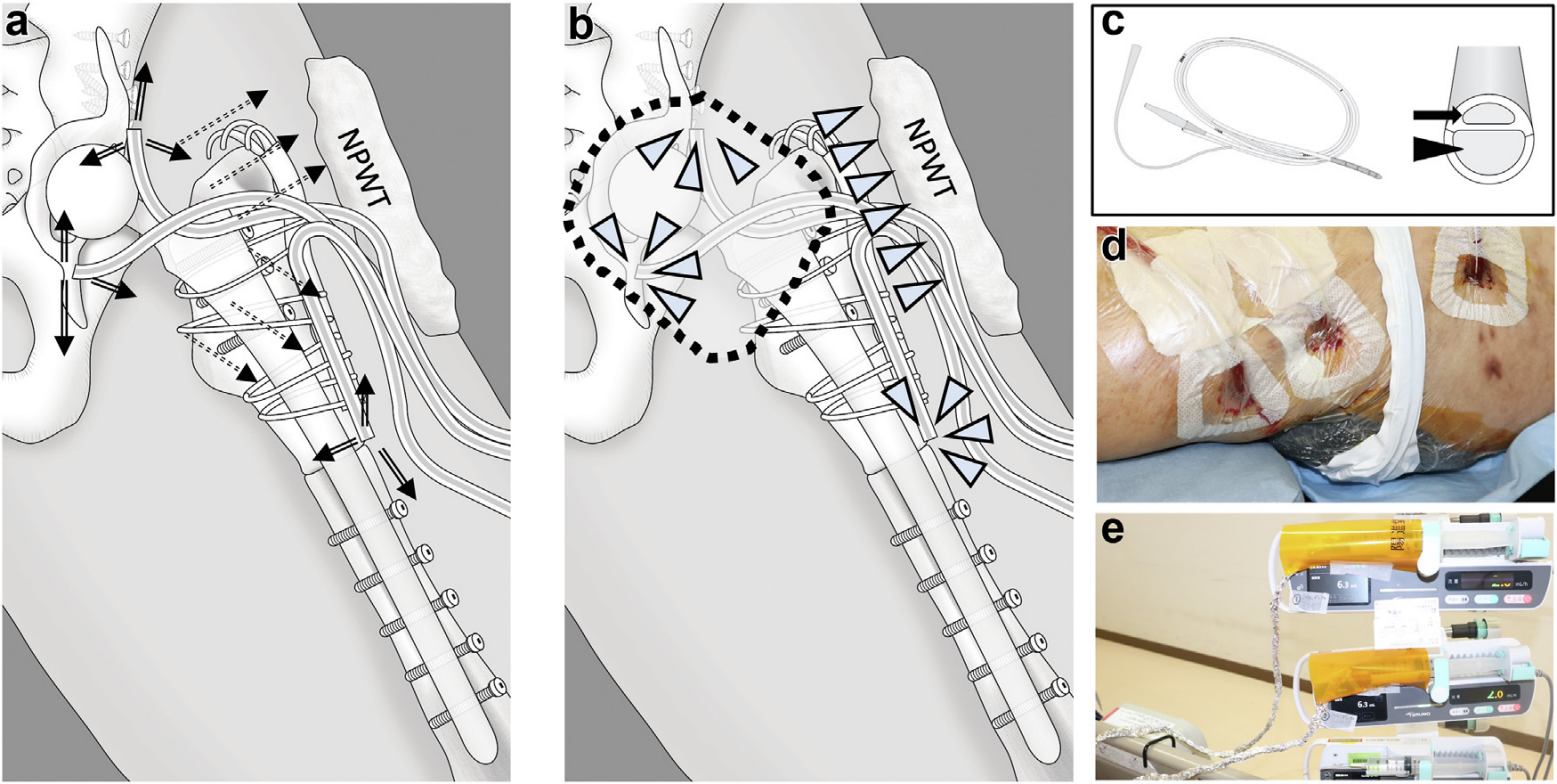
Micafungin (MCFG) was perfused using continuous local antibiotic perfusion (CLAP). (a) Using Salem Sump Tubes, MCFG was directly administrated into infected sites and was diffused (arrows) by negative pressure due to the negative pressure wound therapy (NPWT). (b) Synovial fluid, hematomas, and outdated antimicrobials were continuously suctioned from joint (dash line area) or dead space in soft tissue by NPWT and Salem Sump tubes connected to the NPWT system (arrow head). (c) The double-lumen structure for suction (arrowhead) and MCFG administration (arrow) in the Salem Sump Tube was vital for maintaining continuous perfusion in CLAP. (d) NPWT was used for wound healing in all cases. (e) During the perfusion of MCFG through the CLAP, the syringe and administration route were shielded from light.

The infection area was predicted through computed tomography, magnetic resonance imaging, and scintigraphy for planning antifungal perfusion into the local infectious site of the periprosthetic joint, acetabular and femur, and soft tissues. A bone marrow puncture needle Arrow EZ-IO (Teleflex, Wein, PA) or a bone marrow screw iMAP pin 4 mm in diameter (Cubex Medical, Tokyo, Japan) [16] was used for perfusion for osteolysis in the acetabulum and femur (Fig. 1a-f). If the infection had spread to the pelvis, the iMAP pin was inserted into the iliac crest; if osteomyelitis was present in the femoral bone, an iMAP pin was inserted into the distal part of the femur or greater trochanter or into the iliac crest or femur (Fig. 1e and f). With approval from the institutional review board, a 20-Fr double-lumen Salem Sump Tube (Salem tube) (Japan Covidien, Tokyo, Japan) was inserted into the hip joint or dead space in the soft tissue (iJAP and iSAP, Fig. 1e-h) [16]. The Salem tube was used because it is less likely to clog during perfusion than drainage tubes, and it enables the release of antibiotics to the drainage site (Fig. 2a-c). In all cases, NPWT from RENASYS TOUCH (Smith and Nephew Japan, Tokyo, Japan) was used to promote wound healing, with continuous suction at an average of 50-80 mmHg (Fig. 2d). The NPWT device was also connected to the Salem tube (Fig. 2d) [16]. This enabled continuous drainage of the synovial fluid, hematomas, and outdated antimicrobials in the joints (iJAP) and soft tissue (iSAP) while perfusion of antimicrobial agents into the infected site was continued. The iMAP, iJAP, and iSAP tubes were placed at the center of the infected sites as much as possible, creating a flow path for antimicrobial perfusion into the infected sites and managing dead spaces (Fig. 1).

### Surgery with CLAP

The indication for the surgical procedure was determined based on a guideline from the Infectious Diseases Society of America [2] except 2 cases with chronic fungal PJI who underwent DAIR and CLAP, since the bone defect was expected to be significant if the implant was removed in these cases. A direct lateral approach was used, and the resection of the necrotic tissue was performed. After debridement and/or revision THA, the surgical area was thoroughly cleaned with 3.5% diluted povidone-iodine. Layers of subcutaneous fat, fascia femoris, and gluteus media were reconstructed during wound closure so that the CLAP could perfuse the antifungals all over the infected area. The surgical installment and table were either disinfected or replaced; then, the surgeon changed the gowns and gloves before inserting the iMAP pins or iJAP and iSAP tubes. Soft tissue infection areas were detected using preoperative imaging and intraoperative identification of subcutaneous and intramuscular pocket formation [16]. Usually, 1-2 iJAP tubes are inserted into the hip joints. To increase the amount of the local antifungal administration, 8-Fr Atom multi-purpose tubes (Atom Medical, Saitama, Japan) were inserted into the infection site (Fig. 2).

MCFG (50 μg/mL) was used for the CLAP based on previous studies [[20], [21], [22]]. Using syringe pumps, MCFG (2 mL/h) was administered through each iMAP, iJAP, and iSAP tube [16,17], as well as a multi-purpose tube. During perfusion, the administration route of MCFG was shielded from light using aluminum foil so that the activity of MCFG was not impaired (Fig. 2e). Because all our patients had mixed methicillin-resistant *Staphylococcus* and fungal infections (Table 1), as often seen in clinical situations [23], we administered 1.2 mg/mL gentamicin sulfate (GM) at 2 mL/h into the infection site via CLAP tubes [16] via a different route than MCFG. All patients were also treated with systemic antimicrobials. In addition, serum GM levels were measured weekly during CLAP. We carefully checked the patients for antimicrobial and antifungal side effects.

**Table 1:** Demographics of patients with fungal periprosthetic joint infections.

Case	Age	Gender	Pathogens	History of surgery	Comorbidity	Treatment
1	76	F	*C. albicans, S. epidermidis* (MRS), *S. capitis-capit* (MRS), *Enterococcus spp.*, *Enterobacter aerogenes, Corynebacterium Spp.*, Gram-negative *Bacillus*	11	DDH hyperlipidemia Sick sinus syndrome	Resection arthroplasty with 2 iMAP pins and 2 iJAP tubes. Drug free and able to transfer to wheel chair.
2	72	M	*C. parapsilosis, S. epidermidis, S. caprae* (MRS)	2	HOA hyperlipidemia	2-Staged revision THA with 1 iMAP pin and 2 iJAP tubes during first surgery and 2 iJAP tubes during second surgery.
3	54	M	*C. albicans, S. aureus, S. epidermidis* (MRS), *Kytococcus schroeteri*	8	ONFH diabetes	DAIR with 2 iJAP tubes and 1 iSAP tube.
4	67	M	*C. albicans, S. epidermidis* (MRS), *Corynebacterium Spp*. Gram-negative *Bacillus, Enterobacter cloacae*	3	HOA Infantile paralysis	DAIR with 1 iJAP tube and 1 iSAP tube.

*C.*, *Candida*; DAIR, debridement, antibiotics, and implant retention; DDH, developmental dysplasia of the hip; HOA, hip osteoarthritis; MRS, methicillin-resistant *Staphylococcus*; ONFH, osteonecrosis of the femoral head; *S.*, *Staphylococcus*; *Spp*, *species*.

## Clinical outcome

The clinical outcomes of CLAP in 4 patients who were diagnosed with fungal PJI were investigated, 2 of whom had previously undergone resection arthroplasty, and the other 2 had previously undergone revision THA (Table 1). Of these, 2 patients underwent DAIR and CLAP, and 1 underwent 2-staged revision THA and CLAP. One patient was treated with resection arthroplasty and CLAP (case 1 in Table 1) since this patient had been having significant bone and soft tissue loss due to fungal and multiple bacterial PJI following repeated surgeries. In addition, this patient had been having difficulty controlling fistula formation and a large amount of joint fluid accumulation in the hip joint even after implant removal before CLAP (Fig. 1a and e).

CLAP was administered for an average of 19 days. In all cases, blood GM levels were measured during CLAP, and none of the cases had blood GM levels exceeding 0.3 μg/mL, which was less than the trough concentration for systemic administration. After the removal of CLAP and NPWT from the wound, 3 patients temporarily developed fever and had elevated blood C-reactive protein levels; however, all patients improved rapidly with continued systemic antimicrobial therapy. Three patients who underwent DAIR or revision were able to preserve the allogeneic bones and implants, and 1 patient who underwent resection arthroplasty showed no recurrence of joint fluid accumulation during a mean follow-up of 30 months after surgery (range: 16-54 months). Case 1 required a 4-point walker for a short walk, case 2 achieved full activity in daily life, and cases 3 and 4 required a crutch for long walking with chronic suppression therapy using oral fluconazole at the time of final follow-up.

## Discussion

CLAP is a novel method for directly administering high concentrations of antimicrobial agents at low flow rates into the bone marrow, soft tissues, and joints [16,17]. The concentration of antimicrobials in CLAP can be increased at a targeted concentration that is capable of achieving local destruction of the biofilm [3]; however, the advantages of CLAP have not been reported in cases of PJI and fungal infections. In our study on fungal PJI, 2 patients suffered from septic shock, and all experienced inflammatory symptoms, including joint fluid accumulation, despite systemic antifungal treatment prior to CLAP. Our patients showed rapid resolution of fever and improvement in C-reactive protein levels after CLAP treatment, and no obvious systemic or local antifungal side effects were observed. Echinocandins, an antifungal agent that has been shown to destroy biofilms in basic studies with clinical efficacy and safety, was used for the CLAP therapy [21,[23], [24], [25], [26], [27]]. With sufficient caution, 50 μg/mL MCFG at a flow rate of 2 mL/h showed no side effect in all patients, instead of cytotoxicity in vitro [4,15]. The administration route was shielded with aluminum foil because the MCFG are decomposed by light. Through CLAP treatment with MCFG, patients achieved implant retention, which was vital for ensuring their postoperative daily activity.

One of the advantages of CLAP with antifungal agents is that it can be an additional option to conventional treatments, including antifungal PMMA. Therefore, as observed in case 4, CLAP can also be performed at the time of revision arthroplasty to reduce the possibility of recurrent postoperative infection, along with systemic antibiotic treatment. Another advantage of CLAP is not only local perfusion of antifungal agents but also control of dead spaces by aspirating hematoma formation, especially in cases with severe bone and soft-tissue loss. The NPWT system can be used to manage soft tissue while draining dead space and is expected to be useful in terms of wound management. The disadvantages of this novel treatment are that it requires adequate training in the techniques for local antimicrobial perfusion, knowledge of the necessary supplies and their characteristics, and 2 to 3 weeks of hospitalization. Despite these disadvantages, CLAP can be useful for intractable PJI in combination with conventional surgical treatment in 1- or 2-stage THA cases and implant retention cases.

## Summary

We successfully performed a novel local antifungal treatment, CLAP with MCFG, in patients with fungal PJI. This versatile treatment method can be used in combination with conventional treatment methods for fungal PJI.

## Figures and Tables

**Conflict of Interest Statement for Maruo:**
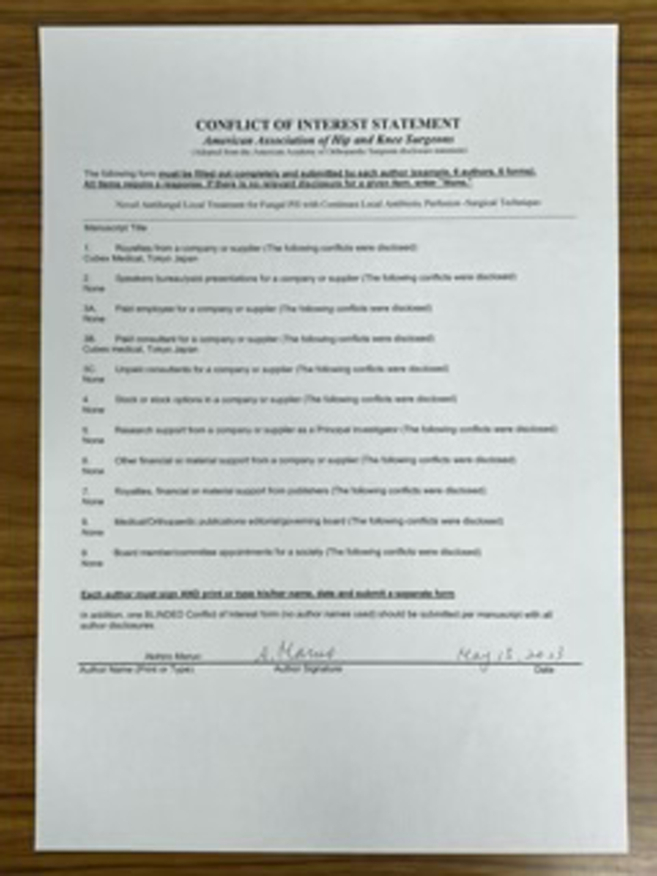


## Appendix A Supplementary data

Conflict of Interest Statement for Abe

Conflict of Interest Statement for Choe

Conflict of Interest Statement for Hieda

Conflict of Interest Statement for Inaba

Conflict of Interest Statement for Kobayashi

Conflict of Interest Statement for Ike

Conflict of Interest Statement for Kawabata

Conflict of Interest Statement for Takeyama
